# Teachers as caregivers of grieving children in school in the post-COVID-19 era: using the self-determination theory to conceptualize teachers' needs when supporting grieving children's mental health

**DOI:** 10.3389/fped.2024.1320106

**Published:** 2024-05-30

**Authors:** Rivi Frei-Landau

**Affiliations:** Faculty of Psychology, Achva Academic College, Arugot, Israel

**Keywords:** self-determination theory, childhood grief, teachers as caregivers, post-COVID-19, qualitative case study research

## Abstract

**Background:**

It has been estimated in recent studies that more than 1.5 million children worldwide lost a caregiver due to the COVID-19 pandemic. Childhood bereavement is associated with heightened risks of impaired academic and social performance, mental health issues, substance use disorders, and higher mortality rates. Yet children may receive insufficient support post-loss. Although the role of school psychologists in supporting grieving students has been examined, little is known about the role of teachers in this context. Specifically, knowledge about teachers' needs when supporting bereaved children is lacking.

**Objective:**

The study's aim was to explore teachers' needs, drawing upon a well-established framework—self-determination theory (SDT)—which focuses on three human needs considered essential for optimal functioning: autonomy, competence, and relatedness.

**Methods:**

Employing a qualitative approach, 36 teachers were interviewed about their needs when supporting grieving students. Interviews were transcribed and then analyzed using thematic content analysis.

**Results:**

Analysis revealed three SDT-related needs: knowledge (theory- and practice-related), acknowledgment, and support (emotional and practical).

**Conclusions:**

The findings enhance our theoretical understanding of childhood bereavement and may promote policy changes that ensure teachers' needs satisfaction. Its significance lies in the basic premise that supporting teachers' needs in the context of pediatric grief may eventually lead to their optimal ability to enact best practices for supporting grieving students' well-being.

## Introduction

Mental health and coping with grief are at the forefront of concerns in the post-COVID-19 era (1–3). Grieving children are particularly vulnerable, as losing a loved one may profoundly impact child development (4). Childhood bereavement is associated with heightened risks of impaired academic and social performance, mental health issues, substance use disorders, and even high mortality rates (5, 6). Yet children are at risk of receiving little to no support post-loss (7). Although the role of psychologists and school counselors in supporting grieving children has been examined (8), research into the role of teachers lags far behind. Particularly, little is known about teachers' needs when dealing with bereaved children. This pilot study examined how teachers' needs were manifested in the context of grieving children by drawing upon a well-established framework—self-determination theory [SDT; (9)]—which focuses on the fulfillment of human needs for optimal functioning. The study's importance lies in the basic premise that understanding teachers' needs can lead to intervention programs that promote teachers' optimal functioning.

### Childhood bereavement and the school context

According to childhood bereavement estimation models in the US, by the age of 18, one in fourteen children (7.2%) will experience the death of a parent or sibling (10). More than 1.5 million children worldwide were estimated to have lost a caregiver during COVID-19 pandemic (11). Relatedly, 70% of American teachers reported having a grieving student in their classroom (12). Yet, the issue of teachers' coping with childhood bereavement has received little academic attention, nor is this issue evident in teacher education formal curricula.

Childhood bereavement studies have mainly focused on teachers' role perceptions or attitudes toward grieving children (13, 14), or toward death education in general (15–18), whereas the issue of teachers' needs has yet to be addressed. Furthermore, although grieving children's needs from schools have been explored (19), teachers' needs have not been investigated, a surprising lack given that gratifying grieving children's needs is assumed to be conditioned upon recognizing and gratifying their caregivers' needs first (20). Although teachers are not expected to provide therapy for their students, they can provide support regarding academic and social issues arising from bereavement, as well as monitor children's mental health. In fact, social support (21–23), acknowledgment (24), and school-based support was argued to facilitate grieving students' adjustment both in the US (25) and Europe (14). Hence, this study aims to fill this knowledge gap by exploring teachers' needs using SDT lens.

### SDT and teachers' needs

Self-determination theory [SDT; (9)] highlights the importance of supporting one's basic psychological needs to ensure optimal functioning. These needs—autonomy, competence, and relatedness—have been found to facilitate high-quality engagement and well-being (26, 27). The need for *autonomy* refers to the experience of authenticity, full willingness, and volition when carrying out an activity; autonomy frustration, by contrast, involves feeling controlled by externally enforced or self-imposed pressures (9). Scholars in the field of teacher education have argued that teacher autonomy may be supported by enabling their choices, encouraging their self-initiation, providing them with a rationale for their work, and acknowledging their perspectives and feelings (27). The need for *competence* refers to one's need to feel effective and capable of realizing objectives (9). Competence frustration involves feelings of doubts about one's efficacy. Therefore, teachers' competence may be supported by a mentor or school principal who communicates a message of trust in the teacher's ability to cope with challenges and provides a clearly structured work plan and assistance in coping with failures when requested (27). The need for *relatedness* comprises an individual's desire to maintain secure and satisfying relationships with others (9). Relatedness satisfaction involves the experience of intimacy and genuine connection with others, whereas relatedness frustration involves the experience of relational exclusion and loneliness. It was argued that teachers’ relatedness may be supported by a mentor or other school figures (e.g., counselors) when these individuals show interpersonal involvement, devote resources and time, and express a willingness to help and a belief in the teacher (27).

Self-determination theory has been previously applied to explore teachers’ needs and the ways these affect their students' learning (28). Kaplan (27) highlighted the necessity of establishing a needs-supportive school environment for teachers during challenging periods. The focus of this study was thus on understanding the aspects that support or hinder the satisfaction of these needs in the context of teachers' coping with grieving children. In line with the SDT premise, it was assumed that supporting teachers’ needs in the context of pediatric grief would eventually lead to their ability to enact best practices for supporting students' optimal functioning.

### Research question

What are teachers' needs in the context of supporting grieving children, and how are these needs manifested in relation to SDT (autonomy, competence, and relatedness)?

## Method

### Study design

Exploring a phenomenon such as teachers’ subjective needs calls for a qualitative methodology, which captures the multifaceted nature of human experience from the individual's standpoint (29). This method is particularly suitable for the study of grief-related issues.

### Participants

The study involved 36 teachers who had a grieving student in their classroom. Teachers' ages ranged from 23 to 64 (8 were young adults—23–30; 9 were 31–40; 8 were 41–50; and 11 were above fifty years old), and their teaching seniority ranged from 1 to 35 years (14 were new teachers with 1–10 years of teaching experience; 6 had 6–20 years of experience; and 16 had above twenty years of teaching experience). Circumstances of the loss were varied: 16 involved anticipated deaths (e.g., cancer), and 20 involved sudden deaths (heart attack/accident/homicide/suicide), as presented in Table 1.

**Table 1:** Demographics of study participants.

Background variables			Frequency *(N* = 36)
Techers’ demographics	Gender	Male	7
		Female	29
	Age	23–30	8
		31–40	9
		41–50	8
		>50	11
	Years of teaching experience	1–10	14
		11–20	6
		>20	16
Bereaved students’ demographics	Age	8–11	13
		12–14	9
		15–18	14
	Education level	Elementary school	13
		Middle school	9
		High school	14
	Circumstances of death	Accident	7
		Anticipated medical reason (e.g., cancer)	16
		Sudden medical situation (e.g., heart attack)	7
		Suicide	5
		Homicide	1
	Years since death	1–3	18
		4–7	12
		8–10	4
		>10	2
	Student's relationship with the deceased	Parent	22
		Sibling	10
		Grandparent	2
		Close friend	2

### Ethics and procedure

The study was approved by the Institutional Ethics Committee of the Academic College (No. 2021_153) [name removed for peer-review]. Written informed consent was obtained from all participants. Participants were told they could withdraw at will. Participants were recruited via snowball sampling (30), an acceptable method in qualitative research. Additionally, messages were posted on social media platforms. Forty teachers expressed an interest in participating, out of whom four eventually declined, deciding that they had no time to be interviewed. After interviewing 36 participants we determined that the saturation criteria had been met (31). Each interview lasted 45–90 min. Interviews were recorded and transcribed. Participant anonymity was ensured by concealing personal information and using pseudonyms in the scientific reports.

### Data collection

In-depth interviews were held by the author and two third-year psychology students who belong to the research lab headed by the author. Interviewers received targeted training by the author, who has extensive experience with qualitative methodology. A two-stage interview procedure, previously used in a qualitative inquiry of loss (32, 33), was used. In the first stage, a general open-ended question was presented. Specifically, participants were asked to reflect about their experiences with grieving students by addressing the following statement/question: “I would like to understand teachers” experiences when dealing with grieving children in their classroom. Can you please share your experience and relate to your needs (as a teacher)? You may begin with whatever feels comfortable to you”. This invitation allowed participants to freely elaborate about whatever they felt was significant in their experience, enabling the interviewer to examine interviewees' spontaneous responses and ask for further elaboration. In the second stage several predefined questions were presented to evoke further elaboration. At the end of each interview, the interviewer confirmed that the interviewee felt well, emotionally.

### Data analysis

Interviews were analyzed using Braun and Clarke's (34) six-phase thematic analysis process.

Specifically, both deductive and inductive analyses were conducted, in a two-step procedure as follows. In the first round, deductive analysis was performed using SDT as an interpretive lens to capture participants' perceived needs. Specifically, the aim was to identify phrases and discourse segments that characterized the participants' accounts of their needs, with a focus on autonomy, competence, and relatedness, while remaining open to possible additional themes that could arise as potential needs. In the second round, an inductive thematic analysis was employed. During this analytic process, data segments that were identified in the previous round as reflecting teachers' needs were analyzed, as we searched for salient themes emerging from the texts. Next, the data were read to identify meaningful units, followed by open coding. During this process, we carefully read and reread the data, to further identify and consolidate relevant themes which would later be conceptualized (35). This careful analysis was conducted to ensure that no important themes were overlooked. It should be emphasized that three independent coders (the author and two research assistants) independently analyzed the initial data, followed by recurrent brainstorming. Cases of disagreement were discussed and settled through conceptual clarification and consensus.

### Trustworthiness

Qualitative inquiry does not traditionally claim to produce “absolute truths” but rather to achieve “trustworthiness” (36). Hence, “investigator triangulation” was performed by the three researchers during coding, analysis, and interpretation of the data. Moreover, “prolonged engagement” was achieved by holding lengthy interviews with the participants, during which trust could be built, and rich and thick data could be produced. Member checking was also conducted, and participants' responses were included in the findings. Finally, the author and interviewers consistently examined their own preconceptions, emotions, and values (i.e., representing the principle of reflexivity) and the ways in which their interpretations or the interviewers' encounters with the participants may have been affected.

### Findings

The qualitative analysis revealed three overarching themes that represent teachers' main needs: the need for knowledge (theoretical alongside practical), the need for acknowledgment, and the need for support. The following excerpts demonstrate these needs. All names used are pseudonyms.

#### The need for knowledge

All participants mentioned their need for knowledge: both theoretical (e.g., how children cope and understand grief) and practical (e.g., how to initiate a fruitful conversation). They also mentioned that there was no clear policy of how to respond, suggesting that this lack of clarity was a barrier preventing the knowledge and guidance they needed in such situations:

There is no tool kit or clear policy on how to deal with such situations. I didn't receive any prior training or guidance about such incidents. So, I felt quite helpless… I didn't feel like I was capable of managing this situation without knowing anything about it… I wasn't sure how to act and I had many dilemmas… Why don't we receive such training as teachers? It's part of life. This is the kind of thing that clearly will happen at some point to one of our students (Rebecca, supporting a student who lost a mother to a heart attack).

As seen, Rebecca felt she needed training, and that this lack of training damaged her ability to feel competent in managing the situation. Josef further elaborated about the types of knowledge he felt he needed:

I needed some training that would include, first of all, knowledge—let's say, about how children typically cope with grief psychologically, how they understand it, what the grief stages or processes are, what I should expect, etc. But I also need to know practically—how to approach him [the grieving student], how to respond in different situations, and so on (Josef, supporting a student who lost a father to suicide).

Josef mentions the need for theoretical alongside practical training. However, when such professional training was not available, some teachers chose to rely on their own experience as a source of knowledge, as conveyed by Noa:

I did not receive any training, but, you know, I personally experienced a loss as a child. So, I felt I knew how to manage it and generally what to expect (Noa, supporting a student who lost a sibling to cancer).

Personal experience with loss not only helped the teachers feel knowledgeable, and thus capable of managing these situations (in theory), but also helped them connect to the grieving child:

I lost my brother as a child, so I felt I was able to connect to her [the grieving child], and I have my own way of looking at it… But this doesn't mean teachers shouldn't be trained for it. I happen to have had my own experience. But what does a teacher who didn't experience loss do?! (Bracha, supporting a student who lost a sibling in a car accident).

In some cases, teachers benefited from the experience of people in their own personal surroundings who had experienced loss:

You may find it funny, but what helped me the most was the fact that my husband is an orphan. I found myself consulting with him about how to act (Dina, supporting a student who lost a father to a disease).

Another aspect mentioned by teachers was the need for formal policies and guidelines regarding the management of such cases. In the face of no clear policy, some teachers received a message they had the freedom to autonomously manage these situations. But unlike the common assumption that freedom to act is considered desirable, in this particular situation—when teachers had no prior personal experience with loss—the lack of policy and formal guidelines made them feel lost and incompetent:

I was told I had the freedom to manage it the way I believed best, but I actually felt the opposite: I wanted guidance and some instruction. I had many dilemmas. Before the funeral, at the *shiva*, throughout the first year. Everything evoked a dilemma. And I was not sure how to manage it and whether I was actually doing it successfully. I felt insecure and exhausted (Dina).

It seems that too much freedom may actually impede teachers' sense of competence as well as their ability to autonomously mange students' grief situations; whereas formal policy would in fact promote teachers' coping.

#### The need for acknowledgment

Teachers mentioned their need to receive acknowledgment of their own emotional turmoil:

It was really difficult. I knew the mother [the deceased] closely and I really was shocked. No one asked me how **I** was doing. I'm expected to function. A teacher should first have a place for herself to process these things. We are human (Lea, supporting a student who lost a mother to an accident).

As appears from Lea, teachers themselves may be grieving. In addition, sometimes teachers' own personal fears can be awakened—an aspect that is overlooked:

It was tough for me. I started having nightmares. Thoughts about my own kids: what would have happened to them if I died. My heart was broken (Tamar, supporting a student who lost a mother to an illness).

As seen, teachers felt they needed to be asked about their own emotions, but this was not acknowledged by school-related personnel, as they are expected to be the ones who function well, regardless. In addition, teachers described their own self-disenfranchisement:

I remember being in such agony when I found out about the death and yet I quickly pushed that aside and started functioning, making sure my student received support… But what about what I needed?! (Inbar, supporting a student who lost a sibling to an illness).

I gave myself ten minutes to cry and then I had to pull myself together and function… but I didn't really give myself the time I needed to process my own grief… I was so alone with it (Vered, supporting a student who lost a close friend).

It appears that acknowledgment is a necessary first step for teachers. Such acknowledgment is also essential for teachers' ability to enact autonomous behaviors when providing support for their students.

#### The need for support

Teachers mentioned their need to receive support from school mental health professionals, such as school counselors or school psychologists, in order to feel “enveloped” and able to personally manage their students' grief situations:

…School was not OK. I should have received support from the school counselor. After all, I was the one supporting the student! But there's no help for teachers in the system. Eventually, I decided to quit (Tamar).

I think that teachers should first receive support prior to supporting their students. I was so attuned to my students and how to support them… **I'm not sure for how long one can support a student without being supported themselves** (Debora, supporting a student who lost a sibling to homicide).

Teachers perceived their role as child-supporters; however, they needed support themselves in order to feel competent to continuously support their students. Not receiving such support was perceived as impeding the support they gave their students:

I felt quite alone there in the situation. **I could have done better had I had some support myself**… I needed some practical advice. But also, I needed some emotional support… I came and said I needed support, but I got the impression that the school psychologist was overloaded with work… We have a teacher meeting on a weekly basis, and usually it's such a waste of time. Instead, they could provide better support to teachers about such situations (Judith, supporting a student who lost a close friend).

Teachers also mentioned that this support should be provided continuously:

I ended up turning to the school counselor, and she did help me. She gave me some advice and instruction. But, you know, it's needed all the time. It doesn't end after the first week. But as a teacher you are sort of left alone with it after the first week (Avital, supporting a student who lost a parent to suicide).

## Discussion

**Figure 1:**
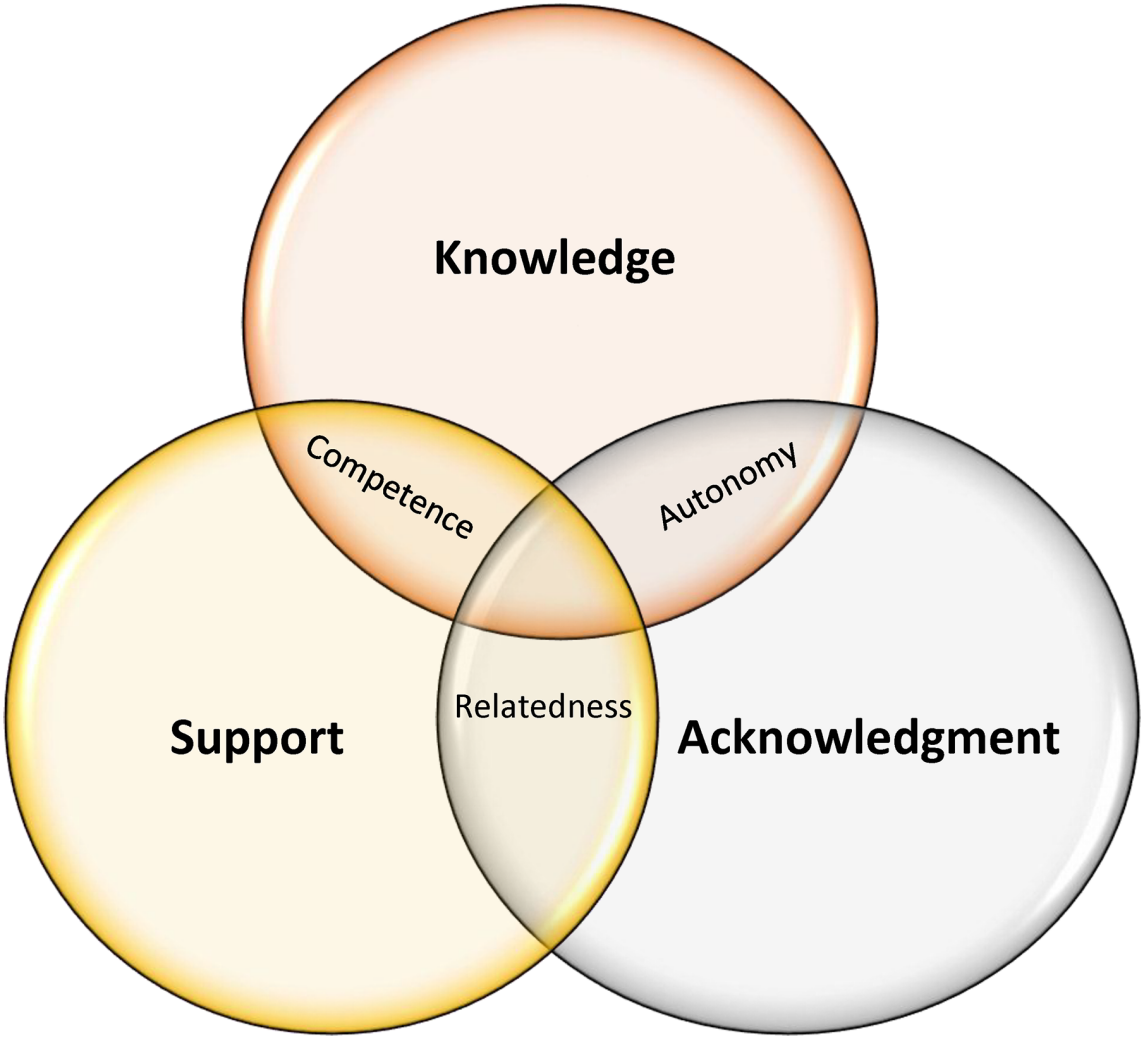
The intersection between teachers’ needs and SDT needs.

The main objective of the current study was to gain an understanding of a previously overlooked phenomenon—teachers' needs in the context of pediatric grief—using the SDT lens. Specifically, it aimed to understand what components enable or impede teachers' sense of autonomy, competence, and relatedness. The study highlighted three main themes representing teachers' needs: knowledge (theory and practice), acknowledgment, and support. Each of these needs was related to two SDT needs. Conceptualizing teachers' needs sheds light on how to optimally attend to teachers’ needs, which may ultimately relate to better outcomes for grieving children. The innovative aspect of this study thus involved its pioneer emphasis on teachers' needs rather than those of school counselors and psychologists, as well as on the use of SDT as a framework to explore teachers' needs rather than students' needs. Figure 1 presents the intersection between teachers' needs and the SDT needs related to them.

### Supporting teachers' *competence* in the context of pediatric grief

As explained, the need for *competence* refers to one's need to feel effective and capable of realizing objectives (9). The study's findings highlight that teachers' sense of competence when supporting grieving children could be achieved by providing them with both *knowledge* and *support*. Knowledge, as perceived by the teachers, consisted of three aspects: theoretical knowledge, practical knowledge, and formal policy. This finding aligns with prior research in the field of teacher education (not in the context of loss), that argued that teachers' competence may be supported by a clearly structured work plan and assistance when requested (27). In this vein, the current study further highlights the necessity for support, knowledge, and clear policy to promote teachers' sense of competence in the context of pediatric grief. Additionally, it echoes previous studies in the field of pediatric grief, advocating for the need to formulate clear school policies (19) and to provide formal teacher training to enable best practices (14).

### Supporting teachers' *autonomy* in the context of pediatric grief

As mentioned, the need for *autonomy* refers to the experience of authenticity when acting (9). The study's findings highlighted that teachers’ sense of autonomy when supporting grieving children may be achieved by providing both *knowledge* and *acknowledgment*. Similarly, in prior research in the field of teacher education (not in the context of loss), it has been suggested that teacher autonomy may be supported by acknowledging their perspectives and feelings and enabling their choices (27). Yet, interestingly, whereas under ordinary circumstances autonomy frustration involves feeling controlled by externally enforced pressures (9), the current study highlights that in the context of pediatric grief, teachers actually seem to crave externally-imposed guidelines (in terms of policy that informs knowledge). In their absence, they seem to feel unable to authentically navigate the mission of supporting their grieving students. This finding is unique and stands in contrast to traditional thinking/perceptions.

### Supporting teachers' *relatedness* in the context of pediatric grief

The need for *relatedness* comprises an individual's desire to maintain satisfying relationships with others (9). In the field of teacher education, teachers' relatedness may be supported by a school figure (e.g., principal, counselor) who show interpersonal involvement, devote resources, and express a willingness to help (27). Indeed, the study highlights that teachers' sense of relatedness when supporting grieving children could be achieved by providing them with both *support* and *acknowledgment*. Both aspects are expected to be provided by school figures such as school mental health professionals. In their absence, the teachers experienced a frustration of their need for relatedness, which prevented them from being able to provide optimal support for their students.

In summary, providing teachers with knowledge, in terms of both theory and practice; acknowledging their struggles; and supporting them throughout the process are all essential for the satisfaction of teachers' needs for competence, autonomy, and relatedness. The fulfillment of such needs will ultimately facilitate their ability to best support their grieving students. These three components should be attended to when designing policy, teachers' training and resources allocation in the context of pediatric grief.

### Limitations and implications

The study's contribution lies in its theoretical insights on the insufficiently explored issue of teachers' needs when dealing with grieving children. Yet it has several limitations. First, knowledge about teachers' coping over time should be gained from longitudinal studies. Second, the transferability of findings may be limited due to the small sample size (*N* = 36) and given that the sample consisted mostly of women. However, we believe that a sample that consists mostly women is adequate considering the overall gender imbalance in the field of teacher education (37). Additionally, in bereavement research, smaller samples are generally deemed sufficient. Future studies should focus on analysis divided into age groups—elementary, middle, and high school. Future studies should attempt to classify and analyze the data in accordance with these abovementioned distinguishing characteristics. That is, since such an approach would allow deeper interpretations drawn from the data. Furthermore, prior experience of personal loss and/or years of teaching experience may have an impact on the three needs identified in the study and, as such, should be investigated. Finally, future research should be conducted among teachers of various cultures and minority groups (38, 39), as participants' backgrounds have been found to be an essential factor affecting their adjustment to stressful situations (40, 41).

In conclusion, this study provides theoretical insights into teachers' needs when supporting grieving children. This topic has received surprisingly little research attention despite grief's potential to profoundly affect school-aged children's mental health. The study has important implications for the theory of childhood bereavement within the context of school-based support; enables a better understanding of the components underlying teachers’ sense of autonomy, competence, and relatedness when supporting grieving children; may improve the design of practical training for teachers; and promotes policy changes that would enable best teacher practices. Finally, the COVID-19 pandemic has highlighted the urgency of exploring the role of teachers in supporting grieving students and the ways in which teachers' needs can be supported (42) to most effectively facilitate the support of children.

## Data Availability

The datasets presented in this article are not readily available because as this is a qualitative research data can not be deposited in order to keep participates’ privacy. It will be available upon demand, with a justified reason, to ensure maximum protection of participants’ anonymity. Requests to access the datasets should be directed to RF, rivipsy@gmail.com.
